# Supplementary material for: OpenMM 7: Rapid development of high performance algorithms for molecular dynamics
Source: PLoS Comput Biol. 2017 Jul 26;13(7):e1005659. doi: 10.1371/journal.pcbi.1005659 (PMC5549999; doi:10.1371/journal.pcbi.1005659)
Supplement: S1 Source Code — (ZIP) [file pcbi.1005659.s002.zip › OpenMM-7.0.1-Source/docs/api-c++/generated/OpenMM.VariableLangevinIntegrator.html]

VariableLangevinIntegrator — OpenMM @OPENMM\_MAJOR\_VERSION@.@OPENMM\_MINOR\_VERSION@ documentation


# VariableLangevinIntegrator¶

*class* `OpenMM::``VariableLangevinIntegrator`¶
:   This is an error contolled, variable time step `Integrator` that simulates a `System` using Langevin dynamics. It compares the result of the Langevin integrator to that of an explicit Euler integrator, takes the difference between the two as a measure of the integration error in each time step, and continuously adjusts the step size to keep the error below a specified tolerance. This both improves the stability of the integrator and allows it to take larger steps on average, while still maintaining comparable accuracy to a fixed step size integrator.

    It is best not to think of the error tolerance as having any absolute meaning. It is just an adjustable parameter that affects the step size and integration accuracy. You should try different values to find the largest one that produces a trajectory sufficiently accurate for your purposes. 0.001 is often a good starting point.

    Methods

    |  |  |
    | --- | --- |
    | `VariableLangevinIntegrator` | Create a `VariableLangevinIntegrator`. |
    | `getTemperature` | Get the temperature of the heat bath (in Kelvin). |
    | `setTemperature` | Set the temperature of the heat bath (in Kelvin). |
    | `getFriction` | Get the friction coefficient which determines how strongly the system is coupled to the heat bath (in inverse ps). |
    | `setFriction` | Set the friction coefficient which determines how strongly the system is coupled to the heat bath (in inverse ps). |
    | `getErrorTolerance` | Get the error tolerance. |
    | `setErrorTolerance` | Set the error tolerance. |
    | `getRandomNumberSeed` | Get the random number seed. |
    | `setRandomNumberSeed` | Set the random number seed. |
    | `step` | Advance a simulation through time by taking a series of time steps. |
    | `stepTo` | Advance a simulation through time by taking a series of steps until a specified time is reached. |

    `VariableLangevinIntegrator`(double *temperature*, double *frictionCoeff*, double *errorTol*)¶
    :   Create a `VariableLangevinIntegrator`.

        |  |  |
        | --- | --- |
        | Parameters: |  |

        - **temperature** – the temperature of the heat bath (in Kelvin)
        - **frictionCoeff** – the friction coefficient which couples the system to the heat bath (in inverse picoseconds)
        - **errorTol** – the error tolerance

    double `getTemperature`() *const*¶
    :   Get the temperature of the heat bath (in Kelvin).

        |  |  |
        | --- | --- |
        | Returns: | the temperature of the heat bath, measured in Kelvin |

    void `setTemperature`(double *temp*)¶
    :   Set the temperature of the heat bath (in Kelvin).

        |  |  |
        | --- | --- |
        | Parameters: |  |

        - **temp** – the temperature of the heat bath, measured in Kelvin

    double `getFriction`() *const*¶
    :   Get the friction coefficient which determines how strongly the system is coupled to the heat bath (in inverse ps).

        |  |  |
        | --- | --- |
        | Returns: | the friction coefficient, measured in 1/ps |

    void `setFriction`(double *coeff*)¶
    :   Set the friction coefficient which determines how strongly the system is coupled to the heat bath (in inverse ps).

        |  |  |
        | --- | --- |
        | Parameters: |  |

        - **coeff** – the friction coefficient, measured in 1/ps

    double `getErrorTolerance`() *const*¶
    :   Get the error tolerance.

    void `setErrorTolerance`(double *tol*)¶
    :   Set the error tolerance.

    int `getRandomNumberSeed`() *const*¶
    :   Get the random number seed. See `setRandomNumberSeed()` for details.

    void `setRandomNumberSeed`(int *seed*)¶
    :   Set the random number seed. The precise meaning of this parameter is undefined, and is left up to each `Platform` to interpret in an appropriate way. It is guaranteed that if two simulations are run with different random number seeds, the sequence of random forces will be different. On the other hand, no guarantees are made about the behavior of simulations that use the same seed. In particular, Platforms are permitted to use non-deterministic algorithms which produce different results on successive runs, even if those runs were initialized identically.

        If seed is set to 0 (which is the default value assigned), a unique seed is chosen when a `Context` is created from this `Force`. This is done to ensure that each `Context` receives unique random seeds without you needing to set them explicitly.

    void `step`(int *steps*)¶
    :   Advance a simulation through time by taking a series of time steps.

        |  |  |
        | --- | --- |
        | Parameters: |  |

        - **steps** – the number of time steps to take

    void `stepTo`(double *time*)¶
    :   Advance a simulation through time by taking a series of steps until a specified time is reached. When this method returns, the simulation time will exactly equal the time which was specified. If you call this method and specify a time that is earlier than the current time, it will return without doing anything.

        |  |  |
        | --- | --- |
        | Parameters: |  |

        - **time** – the time to which the simulation should be advanced

High performance molecular simulation on GPUs


### Navigation

- Core Objects
- Forces
- Integrators
- Extras

©2015, Stanford University and the Authors.
|
Powered by Sphinx 1.3.1
& Alabaster 0.7.7
|
Page source
